# Supplementary material for: Covalently linked dengue virus envelope glycoprotein dimers reduce exposure of the immunodominant fusion loop epitope
Source: Nat Commun. 2017 May 23;8:15411. doi: 10.1038/ncomms15411 (PMC5457521; doi:10.1038/ncomms15411)
Supplement: Supplementary Information — Supplementary Figures [file ncomms15411-s1.pdf]

Supplementary Figures

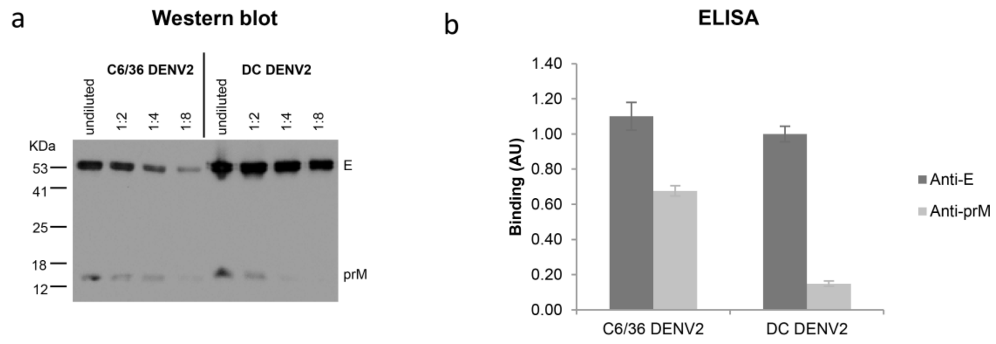

Supplementary Figure 1. prM cleavage in C6/36 and DC produced DENV2

a, The efficiency of PrM cleavage was evaluated by Western blot. C6/36 cell and DC produced virions were run on 12% SDS-PAGE blotted and probed with mouse anti-E mAb (4G2) and human anti-prM (3-147) followed by a cocktail of goat anti-mouse Igs and rabbit anti-human IgG. Finally, the membrane was developed with chemiluminescence substrate.

b, The levels of prM cleavage were also analyzed by detection of E and prM by ELISA. Briefly viral supernatants from C6/36 and DC cells were captured onto plates coated with anti-E mAb (4G2). Then, E and prM were detected by using a humanized version of 3H5 mAb (hu3H5) and human anti-prM (3-147), respectively. Values are shown as mean±SEM from 3 independent experiments.

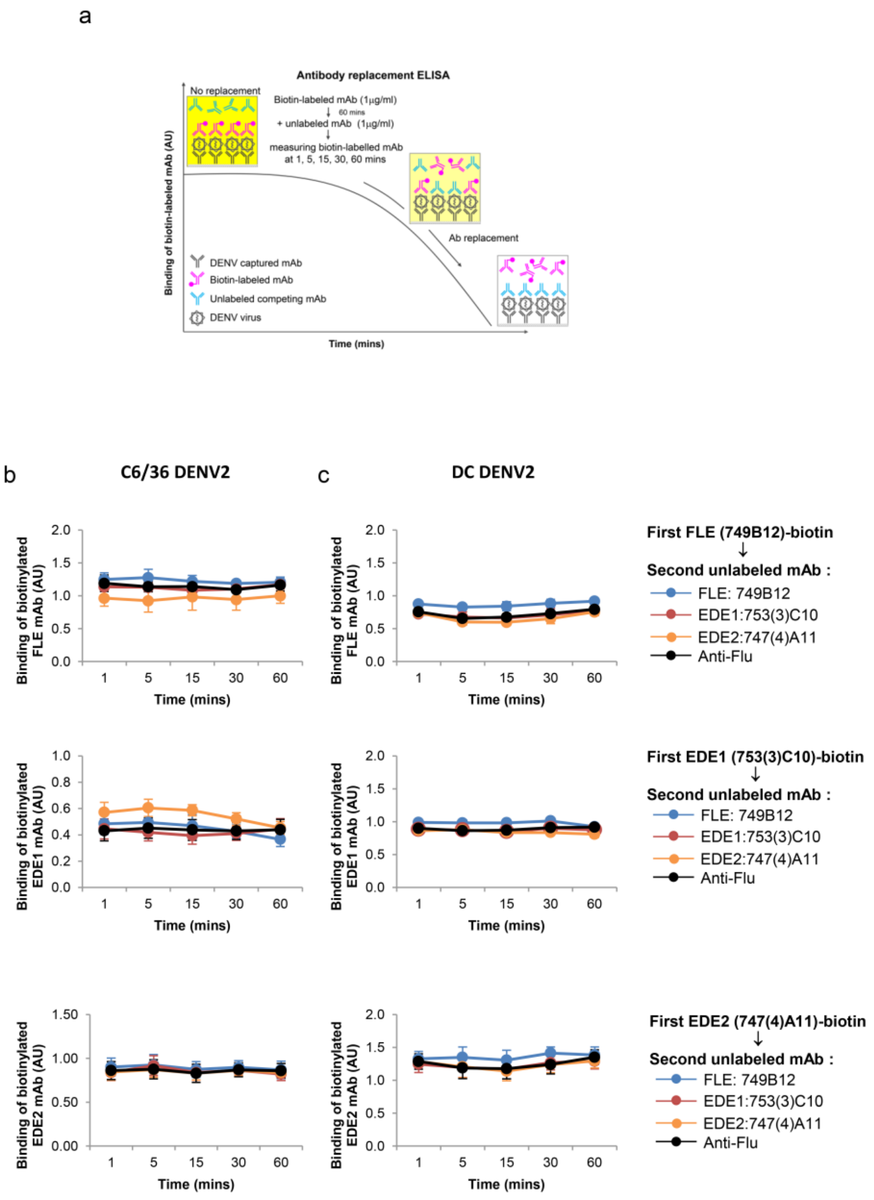

Supplementary Figure 2. Anti-FLE and anti-EDE mAbs stably bound to DENV.
a, Description of the schematic procedure of Antibody replacement ELISA.
b&c, Once bound anti-FLE and anti-EDE mAbs cannot be replaced. Biotinylated antibody was bound to DENV2 particles captured by murine anti-DENV2 EDIII mAb, 2C8, and following washing were incubated with unconjugated competitor antibodies following incubation for the indicated times residual biotin-conjugated mAb was revealed with ALP-conjugated Streptavidin. The data are shown as mean±SEM from 3 independent experiments.

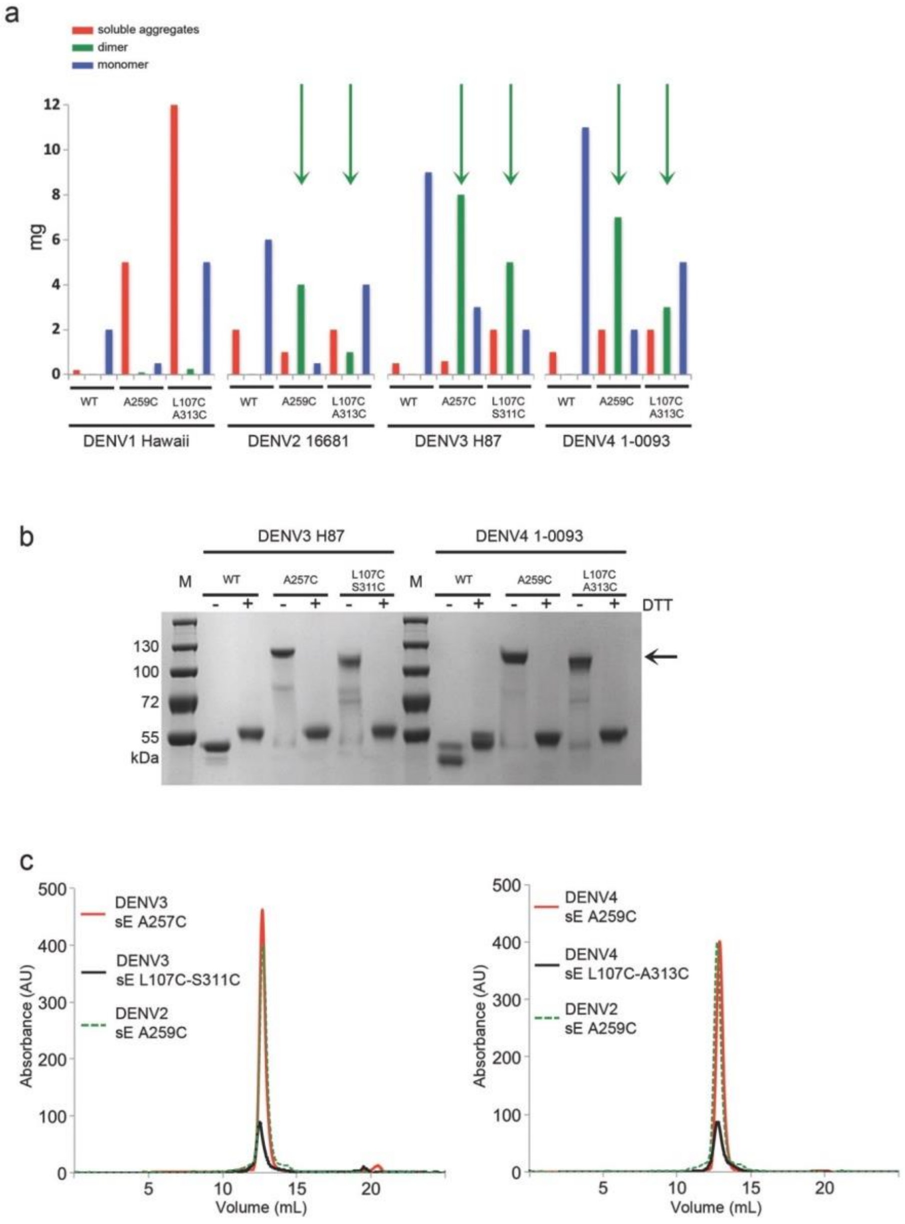

Supplementary Figure 3. Recombinant DENV sE protein yield.

a, Histograms showing approximate yields of monomer, dimer and soluble aggregates during SEC for wild type and two stabilized cysteine mutants of sE from DENV1 Hawaii strain, DENV2 16681 strain, DENV3 H87 strain and DENV4 1-0093 strain are depicted.

b, Coomassie stained SDS PAGE analysis of sE WT and mutants of DENV3 and DENV4 (in absence (-) or in presence (+) of reducing agent DTT). The black arrow on the right indicates the bands of the disulphide stabilized sE dimer.

c, SEC profile of purified single (red trace) and double cysteine (black trace) sE mutants from DENV3 H87 (left panel) and DENV4 1-0093 (right panel) is shown in superimposition to the DENV2 FGA02 A259C mutant (green dotted trace).

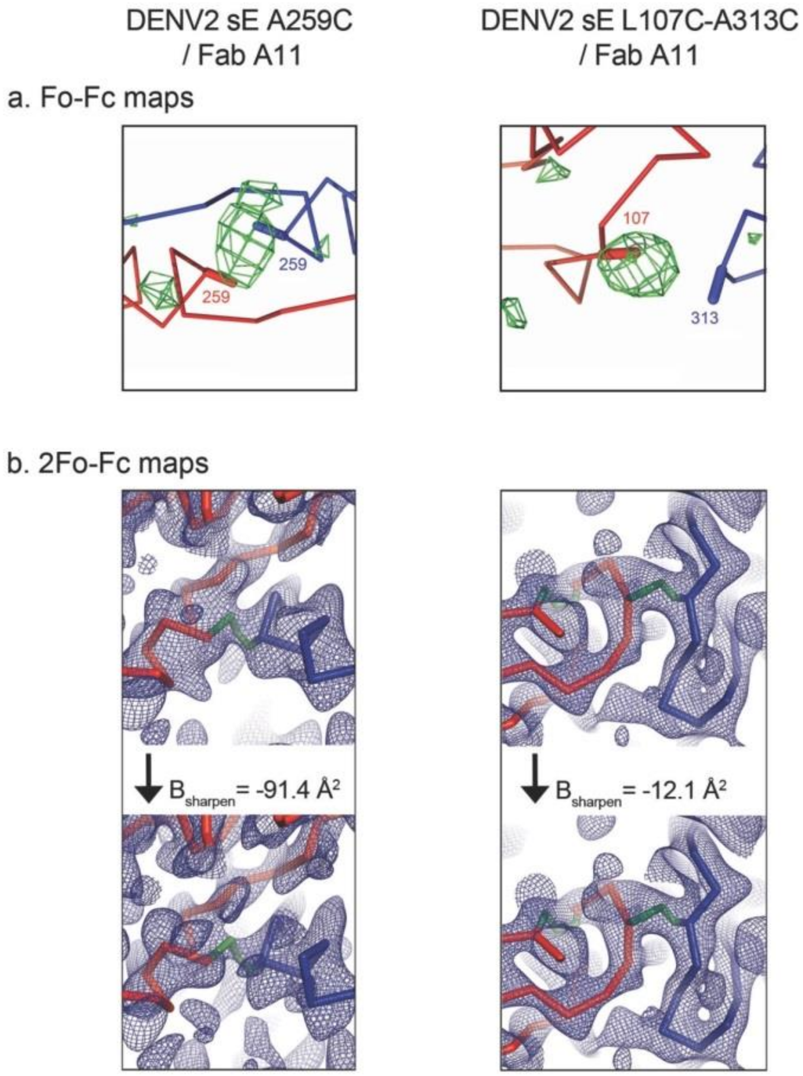

Supplementary Figure 4. Localization of disulphides in DENV2 sE mutants in complex with EDE2 Fab A11.

a,b Difference map around the mutation sites of A259C (left panel) and of L107C/A313C (right panel) structures, calculated from the experimental structure factor amplitudes using phases of the WT 4UTB structure from which the cysteine residues are not present. The green grids represent extra density indicating the presence of disulfides. The peak around residue 259 is observed at 5.6 sigma. The two peaks around residues 107 and 313 in both sE protomers have heights of 6 and 5.1 sigma, respectively.

b, Electron density 2Fo-Fc maps displaying regions around C259, in the A259C sE mutant (left panel on top) and between C107 and C313 in the L107C/A313C sE mutant (right panel on top). Fo and Fc are amplitudes of the structure factures measured and calculated from the final refined model, respectively, for each reflection. The phases are derived from the final model weighed by the agreement between Fo and Fc, and the standard deviation of the measured Fo. Bfactor sharpening applied on the top panels 2Fo-Fc maps are shown in the lower panels for both structures where the engineered Cys positions are depicted in green sticks (see Methods).

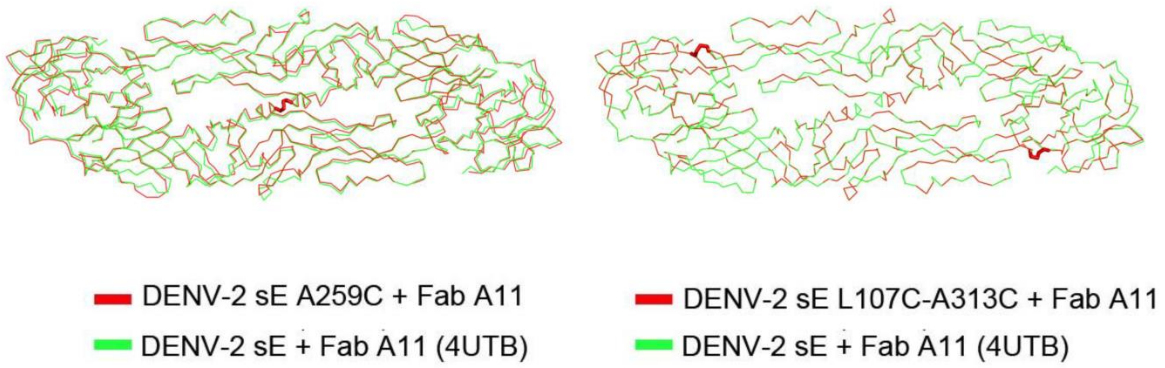

RMSD of aligned E dimer Cα atoms (Å)	DENV2-A259C-Fab A11	DENV2-L107C-A313C-Fab A11
**DENV2-Fab A11 (4UTB)**	1.11 (775)	0.14 (775)

Supplementary Figure 5. Superposition of sE dimers from DENV2 sE WT and from DENV2 sE Cys mutants in complex with Fab A11. Superposition of carbon alpha structures of sE dimer from DENV2 sE WT-Fab A11 structure (4UTB) (shown in green) with sE dimer from DENV2 sE A259C mutant (left panel, in red) and sE dimer from DENV2 L107C/A313C sE mutant (right panel, in red). The table shows the RMSD scores (Å) with the number of Cα atoms used shown in bracket.

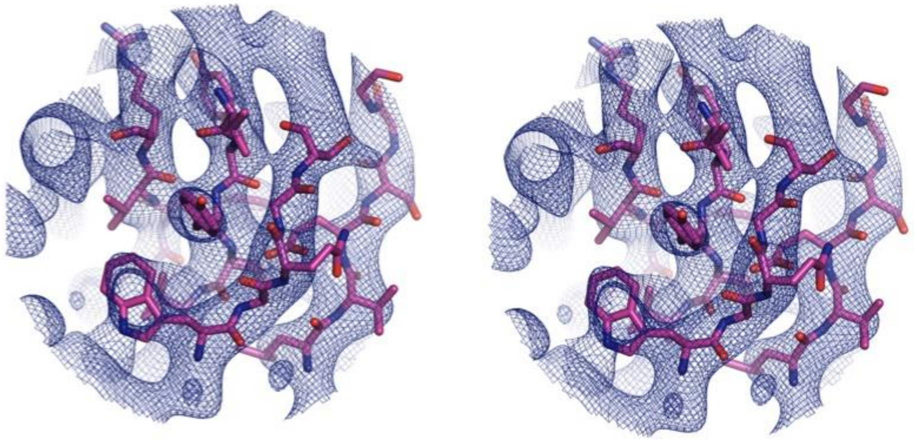

Supplementary Figure 6. Stereo view of electron density map of the DENV2 sE A259C in complex with EDE2 Fab A11. The 2mFo-DFc electron density map (1.0 sigma level) around the Tyr 91 of heavy chain of Fab A11 is shown.
